# Polyphenols isolated from virgin coconut oil attenuate cadmium-induced dyslipidemia and oxidative stress due to their antioxidant properties and potential benefits on cardiovascular risk ratios in rats

**Published:** 2018

**Authors:** Ademola Clement Famurewa, Fidelis Ebele Ejezie

**Affiliations:** 1 *Department of Medical Biochemistry, Faculty of Basic Medical Sciences, Federal University, Ndufu-Alike, Ikwo, Ebonyi State, Nigeria*; 2 *Department of Medical Biochemistry, College of Medicine, University of Nigeria, Enugu Campus, Enugu State, Nigeria*

**Keywords:** Dyslipidemia, Lipid profile, Cadmium, Antioxidants, Polyphenols, Cardiovascular risks

## Abstract

**Objective:**

Literature has confirmed the pathogenic role of cadmium (Cd) and its exposure in the induction of dyslipidemia implicated in the development and increasing incidence of cardiovascular diseases. The current study explored whether polyphenolics isolated from virgin coconut oil (VCO) prevent Cd-induced dyslipidemia and investigate the underlying mechanism of action, in rats.

**Materials and Methods:**

Rats were pretreated with VCO polyphenols (10, 20 and 50 mg/kg body weight; orally) 2 weeks prior to concurrent Cd administration (5 mg/kg) for 5 weeks. Subsequently, serum concentrations of lipid and lipoprotein cholesterol and cardiovascular risk ratios were determined. Hepatic activities of superoxide dismutase (SOD) and catalase (CAT) as well as reduced glutathione (GSH) and malondialdehyde (MDA) contents were analyzed.

**Results:**

Sub-chronic Cd administration significantly increased the serum levels of total cholesterol, triglycerides, low density lipoprotein cholesterol and very low density lipoprotein cholesterol while markedly reduced high density lipoprotein cholesterol. Hepatic activities of SOD and CAT as well as GSH content were suppressed by Cd, whereas MDA level was obviously increased. The co-administration of VCO polyphenol with Cd remarkably restored lipid profile and cardiovascular risk ratios and stabilized antioxidant defense systems comparable to control group.

**Conclusion:**

This is the first study presenting that polyphenols isolated from VCO prevent Cd-induced lipid abnormalities and cardiovascular risk ratios by improving antioxidant defense systems.

## Introduction

Cadmium (Cd) is a widespread environmental and occupational pollutant that has emerged as a major cause of pathobiochemical events and damage in a number of organs. Cadmium concentration has increased in the biosphere through mining, smelting, agricultural and industrial activities (Prozialeck and Edwards, 2012 ▶). It is a stable divalent cation that is not biodegradable and persists in the environment. Human exposures occur in the workplace or via ingestion of Cd-contaminated food or water (Prozialeck and Edwards, 2012 ▶; Vidal et al., 2015 ▶). The Cd levels in the air and drinking water are not alarming; however, a considerable amount of Cd is absorbed via food and cigarette smoking (Siu et al., 2009 ▶). 

Cadmium toxicity represents a classic health hazard due to its numerous deleterious effects in humans and animals. It causes a number of damages in the kidney, liver, testis, pancreas and lung, depending on the exposure dose, duration and route (Larregle et al., 2008 ▶). Several animal studies have indicated that Cd exposure may disturb lipid metabolism, cause dyslipidemia and result in atherosclerosis and diabetes complications (Larregle et al., 2008 ▶; Prabu et al., 2010 ▶; Rogalska et al., 2009 ▶). Cardiovascular diseases (CVDs) are recognized as the leading cause of morbidity and mortality around the world. Altered lipid profile or hyperlipidemia is considered one of the greatest risk factors contributing to the prevalence and severity of atherosclerosis and subsequent coronary heart disease (Zhao et al., 2017 ▶). It has been suggested that the continuous increase in incidence of CVD is due to increased prevalence of exposures to known risk factors, including Cd (Byrne et al., 2009 ▶). Recent epidemiological studies have suggested a positive association between Cd exposure and the incidence and severity of diabetes (Edwards and Prozialeck, 2009 ▶). However, studies have shown that dyslipidemia may potentiate diabetes complications (Tangvarasittichai et al., 2010 ▶). Recently, a study has demonstrated that Cd exposure is associated with hypertriglyceridemia and lower HDL-C, but not with LDL-C or total cholesterol, in residents with Cd exposure (Tangvarasittichai et al., 2015 ▶). In the past two decades, statins have been utilized as the cornerstone of the treatment of dyslipidemia. Although their beneficial effects are appreciated in clinical practice, statins side effects including elevations in liver aminotransferases, neurological damage, myopathy and an increased risk of diabetes have been reported (Zhao et al., 2017 ▶). However, oxidative damage to cell lipid components is suggested as one of the underlying mechanisms for dyslipidemia and pathologies associated with Cd toxicity (Samarghandian et al., 2014 ▶; Samarghandian et al., 2015 ▶). Alteration of lipid metabolism in the liver has been associated with oxidative stress induced by reactive oxygen species (ROS) generated due to Cd toxicity (Samarghandian et al., 2015 ▶). The attainment of possible reduction in coronary heart disease risk and CVD is associated with treatment strategies targeted at reducing the elevated levels of circulatory lipids and lipoprotein components. Recently, alternative medicines are being considered for controlling blood lipids and dyslipidemia.

Current research has shown that natural products may be a promising option to hinder the course of chronic disease development. Accumulating evidence has shown that natural products are rich in bioactive phytochemicals. Recently, there is marked interest in polyphenolic phytochemicals effects on modulating lipid and lipoprotein abnormalities and thereby reducing the risk of CVD (Prabu et al., 2010 ▶). This has led to performing studies on the beneficial potential of dietary nutrients having free-radicals scavenging or antioxidant property to counteract free radicals-mediated cadmium toxicity and reduction in the risk of CVD. One of such dietary nutrient is virgin coconut oil (VCO) which is an oil that is obtained from fresh, mature kernel of the coconut by mechanical or natural means, with or without heating and without using chemical refining (Marina et al., 2009 ▶). Systematic investigations have reported antioxidant properties of VCO in some animal models (Famurewa et al., 2017 ▶; Rahim et al., 2017 ▶). The various beneficial health effects of VCO have been attributed to its potent phenolic compounds conserved by the traditional wet method of production (Illam et al., 2017 ▶). Given the widespread nature of Cd exposure and the emerging role of VCO and its antioxidant properties, extensive investigation is needed to exploit its preventive and therapeutic activity to combat diseases, particularly those related to oxidative stress. Although ROS have been implicated in the pathogenesis of Cd-induced dyslipidemia (Prabu et al., 2010 ▶; Samarghandian et al., 2015 ▶), the protective effect of VCO polyphenol fraction against Cd-induced dyslipidemia remains to be investigated. Thus, this study explored potential effect of VCO polyphenol supplementation on Cd-induced lipid peroxidation, oxidative stress and dyslipidemia in rats. 

## Materials and Methods

**Chemicals**

Cadmium (as cadmium chloride, CdCl_2_), gallic acid, and 1, 1-diphenyl-2-picrylhydrazyl (DPPH) were obtained from Sigma-Aldrich Chemical Co. (St. Louis, MO, USA). Folin-Ciocalteu reagent was purchased from Merck Co. (Darmstadt, Germany). Solvents such as n-hexane and methanol were purchased from Loba Chemie Pvt. Ltd. Mumbai, India. Reagents used for the assays were commercial test kits and products purchased from Randox, UK. All other chemicals used were obtained from local commercial suppliers.

**Animals**

Forty-eight male albino Wistar rats (100-120 g) were used as experimental animals in the present investigation. The rats were housed in animal facility of the Department of Biochemistry, Ebonyi State University, Nigeria, under standard conditions (25^o^C and 12 hr light/12 hr dark cycle). They were kept in cages with wood-chip bedding and allowed free access to standard pellet diet (Vital Feeds Nigeria Ltd, Jos, Nigeria) and clean water, *ad libitum*. The rats were acclimatized one week prior to initiation of the treatment and were handled in a humane manner according to the approved animal experimental procedures of NIH Guidelines for the Care and Use of Laboratory Animals (NRC, 1985 ▶)

**Extraction of VCO**

The coconuts were purchased from the Central market, Abakaliki, Ebonyi State Nigeria, in November, 2016. The VCO was extracted from coconuts (*Cocos nucifera*) according to the method of Nevin and Rajamohan (2004) ▶ as described in a previous study (Famurewa et al., 2017 ▶). Briefly, the viscous slurry obtained from coconut meat (solid endosperm) was prepared as a creamy milky solution by addition of about 400 ml of clean water. The solution was sieved through cheesecloth and the milky filtrate was left for 48 hr to allow the creamy top and water layers separate. The top layer was carefully removed and subjected to mild heating (50^o^C) to remove moisture. The floating oil was gently scooped out and filtered into an air-tight container. This oil which was prepared without refining, bleaching and deodorizing, was used for the isolation of polyphenols administered to rats in the current study.

**Extraction of polyphenols from VCO**

Polyphenols in VCO were extracted according to the method described by Nevin and Rajamohan (2004) ▶ with slight modifications. VCO (10 g) was mixed with 50 mL of n-hexane, and extracted using 20 mL methanol (80%). The process was repeated three times to ensure complete extraction of polyphenols. The three polyphenol fractions were pooled and the extraction solvent was allowed to evaporate. Next, *in vitro* antioxidant activity of the polyphenol fraction was determined.

***In vitro*** **antioxidant assay of polyphenol fraction**

*Determination of total phenolic content*

Total phenolic content of the VCO polyphenol fraction was estimated by Folin–Ciocalteu colorimetric method (Wangensteen et al., 2004 ▶). Measurements were carried out in triplicate and calculations were done based on a calibration curve obtained using gallic acid. The total phenolic content was expressed as milligrams of gallic acid equivalent (GAE) per 100 g oil.

**DPPH free radical scavenging assay**

The ability of the VCO polyphenol fraction to donate a hydrogen atom was assessed by scavenging activity of the 1, 1-diphenyl-2-picrylhydrazyl (DPPH) radical according to the method described by Blois (1958) ▶. The scavenging activity was determined relative to a standard antioxidant, butyrate hydroxytoluene (BHT) and expressed in percentage.

**Ferric reducing antioxidant power (FRAP) assay**

The total antioxidant activity of the VCO polyphenol fraction was evaluated using the ferric reducing antioxidant power (FRAP) assay described by Oyaizu (Oyaizu, 1986 ▶). This assay was based on the reducing power of the potential antioxidant to reduce ferric ions (Fe^3+^) to ferrous ion (Fe^2+^) complex. The reducing power was calculated using the following formula: 

Reducing power (%) = 100 × (absorbance of the control - absorbance of the sample)/absorbance of the control.

**Experimental design**

After 1 week of acclimatization, rats were randomly divided into 6 groups (n=8) as follow:

Group 1 (Normal control): received clean water (1 ml/kg; orally) for 7 weeks.

Group 2 (polyphenols=PF control): received PF (20 mg/kg; orally) for 7 weeks.

Group 3 (Cd control): received Cd (5 mg/kg; orally) for the last 5 weeks of experimental period (Renugadevi and Prabu, 2009 ▶).

Group 4 (PF + Cd I): received PF (10 mg/kg; orally) for 7 weeks + Cd (5 mg/kg; orally,) for the last 5 weeks.

Group 5 (PF + Cd II): received PF (20 mg/kg; orally) for 7 weeks + Cd (5 mg/kg; orally) for the last 5 weeks.

Group 6 (PF + Cd III): received PF (50 mg/kg; orally) for 7 weeks + Cd (5 mg/kg; orally) for the last 5 weeks.

The rats were pretreated daily with polyphenols (10, 20, and 50 mg/kg; orally) 2 weeks prior to concurrent administration of Cd (5 mg/kg) daily for 5 weeks. At the end of the treatment period (7 weeks), all animals (fasted) were slightly anaesthetized using diethyl ether and blood samples were collected from retro-orbital venous plexus by capillary tubes into plain sample bottles and allowed to clot. The clotted blood samples were centrifuged (3000g for 15 min) for separation of serum used for analysis of lipid profile, total cholesterol (TC), triglycerides (TG), low density lipoprotein cholesterol (LDL-C), very low density lipoprotein cholesterol (VLDL-C) and high density lipoprotein cholesterol (HDL-C). The liver was dissected out after sacrifice by decapitation, washed in cold saline solution, dried using tissue paper, homogenized in 0.1M phosphate buffer (1:5 w/v, pH 6.4) and centrifuged (4000 g for 20 min). The obtained supernatant was used for analyses of hepatic antioxidant enzyme activity (SOD and CAT), and GSH and MDA content.

**Biochemical analyses**

Determination of biochemical parameters was carried out using standard commercially available kits purchased from RANDOX, UK. Lipid profile parameters were analyzed in serum stored at 4^o^C using RANDOX kits. The hepatic activity of SOD was assayed by the method of Arthur and Boyne (1985). The activity of catalase was assayed by the method of Sinha (1972) ▶, and GSH level was determined by the method of Exner et al (2000) ▶. Lipid peroxidation was estimated by spectrophotometrically measuring the level of lipid peroxidation product, malondialdehyde (MDA) as described by Wallin et al (1993) ▶. 

**Cardiovascular risk indices**

The coronary risk index (CRI), cardiovascular risk index (CVRI) and atherogenic index (AI) were calculated using the following formulae (Abbott et al., 1988 ▶; Alladi et al., 1989 ▶).

Atherogenic Index (AI) = LDL cholesterol/HDL cholesterol

Coronary Risk Index (CRI) = Total cholesterol/HDL cholesterol

Cardiovascular risk index (CVRI) = Triglyceride/HDL cholesterol

**Statistical analysis**

Results are expressed as mean ± SEM. Statistical evaluation was conducted by one-way ANOVA followed by Tukey’s *post-hoc* test (using SPSS version 22.0 for windows, Inc., Chicago, IL, USA). A p<0.05 was considered significant. 

## Results

**Effects of administration of VCO polyphenols and Cd on lipid profile parameters in treated rats**

Table 1 shows the effect of polyphenols, Cd and their co-administration on serum levels of TC, TG, VLDL-C, LDL-C and HDL-C. The administration of polyphenols (PF) control reduced levels of TC and LDL-C significantly compared to control animals. It was observed that Cd significantly induced dyslipidemia in Cd control rats as evident by marked increases (p<0.05) in serum levels of TC, TG, VLDL-C and LDL-C, as well as significant decreases in HDL-C compared to control levels (p<0.05). Interestingly, it was found that polyphenol administration prior to concurrent Cd administration significantly reversed (p<0.05) the alterations in lipid profile parameters compared to Cd control group. 

**Table 1:** Effects of polyphenols (PF), cadmium (Cd) and their co-administration on serum TC, TG, VLDL-C, LDL-C and HDL-C levels (mg/dl).

**Group**	**TC**	**TG **	**VLDL-C**	**LDL-C**	**HDL-C **
**Control**	4.44 ± 0.16	1.40 ± 0.05	0.70 0.02	1.69 0.15	2.04 ± 0.03
**PF**	4.03 ± 0.05*	1.42 ± 0.02	0.71 0.01	1.25 0.06*	2.04 ± 0.01
**Cd**	4.98 ± 0.17*	1.72 ± 0.03*	0.86 0.02*	2.16 0.22*	1.86 0.06*
**PF + Cd I**	4.25 ± 0.09#	1.54 ± 0.01#	0.77 ± 0.01#	1.46 ± 0.10#	2.02 ± 0.03#
**PF + Cd II**	4.29 ± 0.08#	1.52 ± 0.03#	0.76 ± 0.02#	1.54 ± 0.08#	2.00 ± 0.04
**PF + Cd III**	3.98 ± 0.08#	1.50 ± 0.01#	0.75 ± 0.01#	1.16 ± 0.07#	2.07 ± 0.01#

Results were expressed as mean ± SEM (n=8). A p<0.05 was considered significant.

* p<0.05: the difference is significant compared to control group in the same column.

# p<0.05: the difference is significant compared to Cd group in the same column.

**Effects of administration of VCO polyphenols and Cd on coronary and cardiovascular risk indices in treated rats**

Table 2 depicts the effect of VCO polyphenols, Cd and their co-administration on atherogenic index (AI), coronary risk index (CRI) and cardiovascular risk index (CVRI) in treated rats. Cadmium administration to rats significantly increased AI, CRI and CVRI compared to control. VCO polyphenols (10, 20 and 50 mg/kg) co-administration with Cd (5 mg/kg) prominently decreased AI, CRI and CVRI in comparison to Cd control group. However, VCO polyphenols only markedly reduced AI and CRI compared to control group.

**Table 2:** Effects of polyphenols (PF), cadmium (Cd) and their co-administration on atherogenic index (AI), coronary risk index (CRI) and cardiovascular risk index (CVRI) in treated rats.

**Group**	**AI**	**CRI**	**CVRI**
**Control**	0.83 ± 0.07	2.16 ± 0.07	0.68 ± 0.03
**PF**	0.61 ± 0.03*	1.97 ±0.02*	0.69 ± 0.01
**Cd**	1.19 ±0.15*	2.71 ± 0.15*	0.93 ± 0.03*
**PF + Cd I**	0.73 ±0.06#	2.11 ±0.06#	0.76 ± 0.01#
**PF + Cd II**	0.78 ± 0.05#	2.16 ±0.06#	0.77 ±0.02#
**PF + Cd III**	0.56 ± 0.04#	1.92 ± 0.04#	0.73 ±0.01#

Results were expressed as mean ± SEM (n=8). A p<0.05 was considered significant.

* p<0.05: the difference is significant compared to control group in the same column.

# p<0.05: the difference is significant compared to Cd group in the same column.

**Antioxidant activity of polyphenol fraction**

Table 3 shows the estimation of total phenol content, DPPH scavenging activity and ferric reducing antioxidant power of methanol-extracted polyphenol fraction.

**Effect of administration of VCO polyphenols, and Cd as well as their co-administration on hepatic markers of oxidative stress in treated rats**

**Figure 1:**
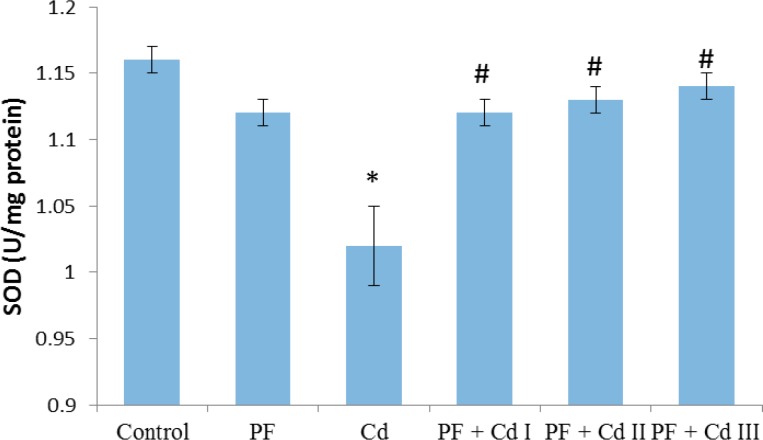
Effect of administration of polyphenols (PF), and Cadmium (Cd), as well as their co-administration on hepatic superoxide dismutase (SOD) activity in Cd-treated rats. Results were expressed as mean ± SEM (n=8). A p<0.05 was considered significant. *p<0.01: significant when compared to control group; ^#^p<0.01: significant when compared to Cd group

**Figure 2:**
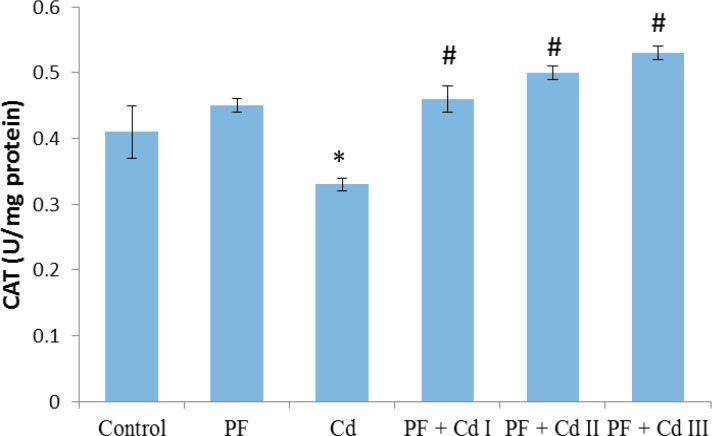
Effect of administration of polyphenols (PF), and cadmium (Cd) as well as their co-administration on hepatic catalase (CAT) activity in Cd-treated rats. Results were expressed as mean ± SEM (n=8). A p<0.05 was considered significant. *p<0.01: significant when compared to control group; ^#^p<0.01: significant when compared to Cd group

**Figure 3:**
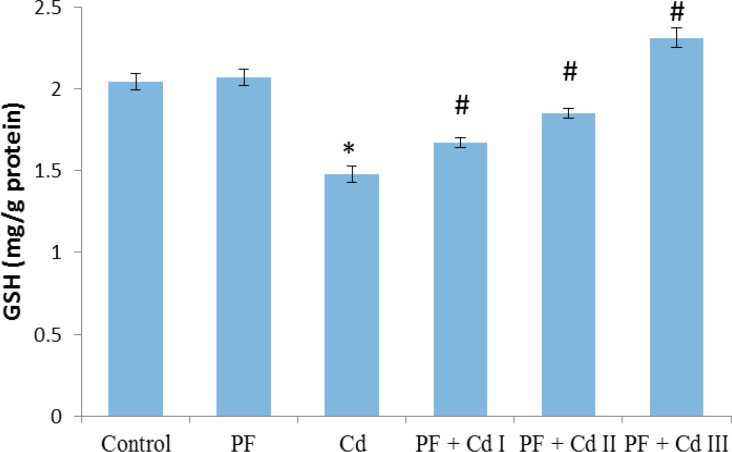
Effect of administration of polyphenols (PF), cadmium (Cd) as well as their co-administration on hepatic glutathione (GSH) content in Cd-treated rats. Results were expressed as mean ± SEM (n=8). A p<0.05 was considered significant. *p<0.01: significant when compared to control group; ^#^p<0.01: significant when compared to Cd group

**Figure 4:**
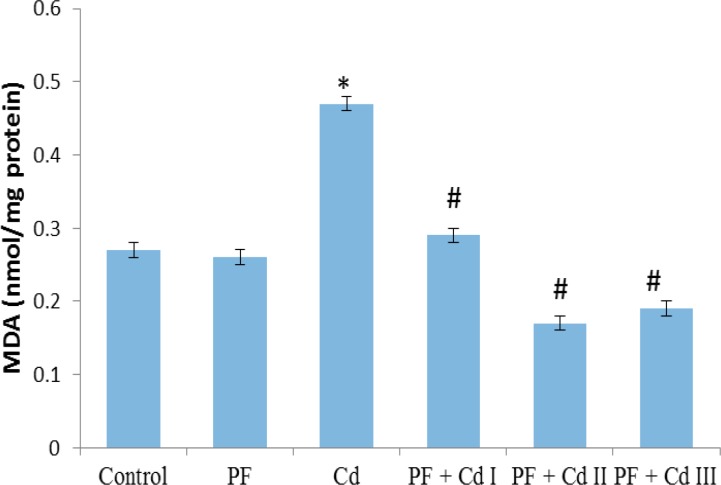
Effect of administration of polyphenols (PF), cadmium (Cd) as well as their co-administration on hepatic malondialdehyde (MDA) content in Cd-treated rats. Results were expressed as mean ± SEM (n=8). A p<0.05 was considered significant. *p<0.001: significant when compared to control group; ^#^p<0.01: significant when compared to Cd group

The hepatic markers of oxidative stress are depicted in Figure 1, 2, 3 and 4. It was observed that sub-chronic administration of Cd (5 mg/kg) to rats significantly decreased (p<0.05) the hepatic activities of SOD and CAT, as well as hepatic content of non-enzymatic antioxidant, GSH, compared to control group (Figures 1-3). Lipid peroxidation marker, MDA, in the hepatic tissue was significantly increased (p<0.05) in rats treated with Cd only (Figure 4). The levels of the afore-mentioned markers were comparable to control in rats administered with polyphenols (20 mg/kg) only. However, the administration of polyphenols (10, 20 and 50 mg/kg) prior to and along with cadmium exposure in groups PF + Cd I, II and III remarkably reduced oxidative stress and lipid peroxidation markers compared to Cd group (p<0.01) and their levels were comparable to normal control rats.

**Table 3:** Total phenol content, DPPH scavenging activity and ferric reducing antioxidant reducing power (FRAP) of methanol-extracted polyphenol fraction

**Total phenol content (mg GAE/100g)**	**DPPH scavenging activity (%)**	**FRAP (%)**
57.11 ± 0.05	68.24 ± 0.10	48.96 ± 0.29

Values represent mean ± SEM of triplicate analysis. DPPH: 1, 1-diphenyl-2-picrylhydrazyl.

## Discussion

Cadmium is a non-essential heavy metal known to induce a broad spectrum of toxicological effects and biochemical disruptions posing serious hazards to health. Published papers indicate that Cd toxicity triggers lipid and lipoprotein abnormalities which play undeniable roles in the pathogenesis of CVD and diabetes complications (Samarghandian et al., 2015 ▶). The consequent morbidity and mortality from these diseases have a substantial socioeconomic impact and reduce the quality of life. The search for dietary-based strategies to prevent dyslipidemia besides pharmacological approaches such as statin therapy is still an area of ongoing research.

In the present study, sub-chronic Cd administration induced lipid abnormalities as evident by considerable increases in serum levels of TC, TG, LDL-C, and VLDL-C associated with a significant decrease in HDL-C (Table 1). The findings show that Cd exposure indeed alters metabolism of lipids corroborating the reports of earlier studies indicating that Cd triggers dyslipidemia that may results in atherosclerosis (Prabu et al., 2010 ▶; Tangvarasittichai et al., 2015 ▶; Samarghandian et al., 2015 ▶). Although the kidney and liver are the primary targets of Cd toxicity, the liver is the major site of lipid and lipoprotein metabolism and hepatotoxic effect of Cd on liver is well reported (Vincent-Sánchez et al., 2008 ▶; Renugadevi and Prabu, 2010 ▶). It is accepted that heavy metals affect 3-hydroxy-3-methylglutaryl CoA reductase (HMG-CoA reductase) activity which alters cholesterol synthesis and lipid metabolism (Senthilkumar et al., 2012 ▶; Zhou et al., 2016 ▶). However, the underlying pathogenic mechanism of dyslipidemia induced by Cd exposure remains to be fully understood (Zhou et al., 2016 ▶). Nevertheless, the findings of experimental studies show that Cd in particular, directly inhibits numerous enzymes related to lipid metabolism (Ramirez and Gimenez, 2002 ▶; Larregle et al., 2008 ▶; Zhou et al., 2016 ▶). Research reports found that Cd administration increases the activity of HMG-CoA reductase in the plasma and liver tissue (Prabu et al., 2010 ▶; Bashir et al., 2014 ▶). HMG-CoA reductase is a rate-limiting enzyme in cholesterol biosynthesis. An increase in the activity of HMG-CoA reductase increases the biosynthesis of cholesterol generally in hepatic cells and this may significantly contribute to alterations in lipid compounds levels in tissues and circulation (Larregle et al., 2008 ▶; Prabu et al., 2010 ▶; Rogalska et al., 2009 ▶). Furthermore, Cd has been linked to excess free fatty acid (FFA) in circulation which promotes conversion of FFA into phospholipids and cholesterol in the liver Senthilkumar et al., 2012 ▶; Belyaeva and Korotkov, 20030 ▶). The formation of phospholipids and cholesterol with excess TG in the liver is released into the circulation (Mayes and Botham, 2003 ▶) The results of the current study are in consonance with previous reports on cadmium potential in causing lipid and lipoprotein abnormalities (Tangvarasittichai et al., 2015 ▶; Samarghandian et al., 2015 ▶; Ramirez and Gimenez, 2002 ▶; Bashir et al., 2014 ▶; Zhou et al., 2016 ▶). The VCO polyphenols administration along with Cd reversed the lipid abnormalities induced by Cd (Table 1). Consistent with our results, previous research indicated that a number of polyphenols from natural products have beneficial effects against dyslipidemia (Prabu et al., 2013 ▶; Danavi et al., 2015 ▶) 

However, aside from Cd effect on enzymes involved in lipid metabolism, oxidative stress appears to be the second category in the landscape of Cd-associated dyslipidemia. As pointed out by Olisekodiaka et al (2012) ▶, lipid peroxidation induced by Cd may play a role in the pathogenesis of lipid abnormalities. The authors found that Cd-induced dyslipidemia was considerably associated with reduced total antioxidant status, suggesting oxidative stress role. Serum levels of lipid peroxidation marker, MDA, found to be markedly raised along with reduced level of GSH in a rat model of Cd-induced dyslipidemia (Samarghandian et al., 2015 ▶). Furthermore, in the current study, hepatic activity of oxidative stress markers, SOD and CAT, as well as GSH content were prominently reduced in Cd-treated rats (Figures 1, 2 and 3). In agreement with the previous work (Olisekodiaka et al., 2012 ▶; Samarghandian et al., 2015 ▶), we found that hepatic lipid peroxidation was significantly high as evident by MDA levels (Figure 4). Considering the oxidative mechanism by which Cd induces oxidative stress, it is conceivable to suggest that oxidative stress may contribute to dyslipidemia development. Cd is well reported to induce oxidative stress by producing hydroxyl radicals, superoxide anions, hydrogen peroxide and nitric oxide via indirect mechanisms of depleting GSH level, and reducing SOD, CAT and other antioxidant enzyme activities (Vincent-Sánchez et al., 2008 ▶). In fact, a study has suggested that enhanced activity of HMG-CoA reductase is associated with increased lipid peroxidation in Cd-treated rats (Bashir et al., 2014 ▶). Depletion of GSH precedes lipid peroxidation and atherogenesis *in vivo* (Covas et al., 2006 ▶). SOD and CAT are important antioxidant enzymes that mitigate oxidative stress *via* ROS elimination. SOD is involved in the dismutation of superoxide radical to hydrogen peroxide (H_2_O_2_), while CAT decomposes H_2_O_2_ to water and molecular oxygen (Fakurazi et al., 2012 ▶). The Cd toxicity in decreasing the hepatic GSH and antioxidant enzymes (SOD and CAT), observed in our study, has been already reported (Vincent-Sánchez et al., 2008 ▶). Evidently, the enhanced lipid peroxidation in Cd-treated rats is associated with depressed activity of antioxidant enzymes. Our observations therefore suggest that Cd intoxication may compromise hepatic antioxidant defense system to favor oxidative stress development linked to lipid abnormalities. Administration of VCO polyphenols before and along with Cd notably increased GSH content and activities of SOD and CAT while decreased MDA level in the liver of rats comparable to the normal rats. The observed improvement may be due to the potent antioxidant and membrane-stabilizing properties of VCO polyphenols. Dietary phenolic compounds are natural antioxidants reputed as potent free-radical scavengers in biological systems and improve cellular antioxidant defense systems (Vincent-Sánchez et al., 2008 ▶). Our findings herein corroborate the recent experimental studies showing antioxidant potency of VCO polyphenolics as demonstrated by improvement in lipid peroxidation and antioxidant enzyme activity in an experimental model of arthritis and colon epithelial cell line (Vysakh et al., 2014 ▶; Illam et al., 2017 ▶). 

Moreover, evidence shows that lipid ratios are important in predicting cardiovascular risk (Gasevic et al., 2014 ▶). Evaluation of lipid ratios is currently being considered to optimize the predictive capacity of the lipid profile in risk of CVD (Millán et al., 2009 ▶; Gasevic et al., 2014 ▶). Studies have shown that LDL-C/HDL-C (AI) and TC/HDL-C (CRI) ratios are the strongest determinants of CVD risk (Grundy et al., 1989 ▶). In the present study, oral administration of VCO in concomitant exposure to Cd showed significant reductions in the atherogenic index (AI), coronary risk index (CRI) and cardiovascular risk index (CVRI) compared to Cd-treated group (Table 2). CRI is a more powerful coronary risk predictor than independently used total cholesterol, LDL cholesterol and HDL cholesterol of coronary heart disease risk, and designated TC/HDL cholesterol (Ingelsson et al., 2007 ▶). According to Vega et al (2014) ▶, CVRI has been associated with cardiac disease mortality. Based on these findings, polyphenols from VCO are potentially beneficial supplements for improving lipid and cholesterol profile and cardiovascular risk. Perceptibly, it may be difficult or even impossible to eliminate Cd from the environment because of the continued worldwide anthropogenic mobilization and increased Cd production and consumption in industries (Zhou et al., 2017 ▶). Statin therapy is the cornerstone of dyslipidemia management; patients often discontinue these medications abruptly because of their side-effects (Danavi et al., 2015 ▶). But the desirable health promoting properties of polyphenols have low or no side effects on biological processes. Exposure to environmental toxicants such as Cd, a source of oxidative stress and lipid abnormalities, may be managed by using dietary antioxidant phenolic compounds. 

Conclusively, this is the first study to assess beneficial health effect of polyphenols extracted from VCO in Cd-induced dyslipidemia. This study confirmed previous findings that Cd can adversely affect lipid and lipoprotein profile. It has provided evidence that dyslipidemia induced by Cd exposure may be attenuated by antioxidant property of VCO polyphenols in rats. 

## Conflict of interest

The authors declare no conflict of interest
